# The need for neonatal jaundice screening awareness in the Pakistani population: short communication

**DOI:** 10.1097/MS9.0000000000000960

**Published:** 2023-07-03

**Authors:** Hafsa Naeem, Kaleem Ullah, Sidhant Ochani, Khadija Naeem, Hafiz B. Ahmad, Md. Al Hasibuzzaman

**Affiliations:** a Department of Medicine, Dow International Medical College, Karachi; b Department of Liver Transplantation, Pir Abdul Qadir Shah Jeelani Institute of Medical Sciences, Gambat; c Department of Medicine, Khairpur Medical College, Khairpur Mir’s, Pakistan; d Institute of Nutrition and Food Science, University of Dhaka, Dhaka, Bangladesh

**Keywords:** awareness, neonatal health, neonatal jaundice, Pakistan, screening

## Abstract

Neonatal jaundice is a common illness that affects around 80% of preterm and 50–60% of full-term newborn infants. It is one of the most common causes of neonatal death. Neonatal jaundice may be physiological or pathological. Physiologic jaundice is far more common than pathologic jaundice and accounts for most hyperbilirubinemia. Physiologic jaundice in neonates is due to greater hemoglobin breakdown compared to bilirubin clearance. While pathological jaundice occurs due to various infections, drug toxicity, inborn enzyme deficiencies, Rhesus fetal-maternal incompatibility, hypothyroidism, and congenital biliary duct obstruction diseases. In many parts of the world, midwives, and nurses perform spontaneous vaginal deliveries and they only rely on visual screening for neonatal jaundice. However, this is not reliable, especially for newborns having darker skin. Educating the mothers on screening for early detection of neonatal jaundice and seeking medical treatment in a country like Pakistan, which is considered a high-risk population, is crucial. Also, as most females give birth at home, hence, midwives’ knowledge about neonatal jaundice also needs to be improved.

A serum bilirubin level of greater than 5 mg/dl in a neonate is called neonatal jaundice. Neonatal jaundice is a common illness that affects around 80% of preterm and 50–60% of full-term newborn infants. It is one of the most common causes of neonatal death^1^. Neonatal jaundice may be physiological or pathological. Physiologic jaundice is far more common than pathologic jaundice and accounts for most hyperbilirubinemia. Physiologic jaundice in neonates occurs due to more hemoglobin breakdown compared to bilirubin clearance. While pathological jaundice occurs due to various infections, drug toxicity, inborn enzyme deficiencies, Rhesus fetal-maternal incompatibility, hypothyroidism, and congenital biliary duct obstruction diseases^2^.

The first week after childbirth is critical for diagnosing and monitoring neonatal jaundice. For the initial assessment, the physicians look at the neonatal skin and sclera color along with the neurological examination and rule out the cephalo-hematoma, and organomegaly. In many parts of the world, midwives, and nurses perform spontaneous vaginal deliveries and they only rely on visual screening for neonatal jaundice. However, this is not reliable, especially for newborns having darker skin^1^. Every neonate with rapidly progressive or persistent jaundice should undergo a detailed workup to rule out the pathological causes of neonatal jaundice. In addition to this visual screening, a local study suggested that urinalysis should also be included in the workup for neonates with hyperbilirubinemia, especially for those neonates having a surge in the unconjugated bilirubin^1^. The treatment for neonatal jaundice includes phototherapy, where blue-green spectrum (wavelength 460–490 nm) light is used to convert unconjugated bilirubin into bilirubin photoproducts in the skin, which the body can metabolize. Sunlight or specialized commercial light is used for this purpose. Phototherapy’s success rate in treating neonatal jaundice is remarkable and nearly 100%.

Severe bilirubinemia can have grave consequences, especially during the first week. Bilirubin crosses the blood-brain barrier during the first week and a high bilirubin level may cause severe brain damage and various neurological complications, that is, acute bilirubin encephalopathy and kernicterus. Kernicterus is a dangerous condition that manifests as athetoid cerebral palsy, a movement disorder characterized by abnormal involuntary movement, hearing loss, speech impairment, and other intellectual disabilities, which are usually permanent^3^.

In developed countries like the United States, complications due to neonatal jaundice are fairly small because of good surveillance and prompt treatment due to easier access to specialized health facilities. However, in a developing country like Pakistan, a significant proportion of neonates are born with low birth weight, which is a major predisposing factor for developing neonatal jaundice. In rural Pakistan, women mostly opt for home deliveries by midwives. Also, due to early discharge from the hospital after the delivery, the diagnosis of severe neonatal jaundice is often missed^4^. In Pakistan, mothers are usually discharged after one or two days of hospital stay. Additionally, East Asians already have higher baseline neonatal bilirubin levels^5^. All these factors predispose neonates in the Pakistani population to develop early severe hyperbilirubinemia and its related complications.

A study in Pakistan in 2017 evaluated 255 cases of neonatal jaundice retrospectively. The mean age of appearance of jaundice in this study population was 2.48±1.32 days; 52.9% of these neonates were found to be underweight (<2.5 kg), 50.2% were preterm (<37 weeks), and 16% were found anemic. Out of all, 8.6% of neonates progressed to kernicterus. The study population frequently found various predisposing factors for developing neonatal jaundice. The key fact to note in this study was the relationship between low birth weight and increased incidence of developing kernicterus^6^.

Another study from a remote area in Pakistan used a questionnaire to assess women’s knowledge, attitude, and practice toward neonatal jaundice. Out of 107 women, only a small number of participants (37.38%) were aware of neonatal jaundice. It was alarming to note that only 11.21% of participants heard about neonatal jaundice from a physician, indicating a lack of interest on the clinicians’ behalf. At the same time, most of the respondents’ sources of information were old-aged family members despite being conducted in a rural area, where people often disregard medical advice or treatment due to financial constraints or hesitancy towards acceptance of medical treatment. Still, 83.9% of the participants were positive about seeking medical consultation^4^.

Educating the mothers on screening for early detection of neonatal jaundice and seeking medical treatment in a country like Pakistan, which is considered a high-risk population, is crucial. Also, as most females give birth at home, hence, midwives’ knowledge about neonatal jaundice also needs to be improved.

The primary source of medical information should be a physician, and it should be mandatory for physicians and nurses to educate mothers on different neonatal danger signs for early detection and prompt treatment. Researchers have developed a mobile application to screen neonatal jaundice following an initial pilot study on 37 newborns in 2020. The same application was used in Ghana, where screening was done on 336 newborn babies. Seventy-nine babies were reported to have severe jaundice in real, and the app recognized 74 cases. The results of the applications were also compared with the standard device transcutaneous bilirubin meter, which identified 76 cases of severe neonatal jaundice. The results of both devices were comparable^7^. This application or an alternate locally developed application in the local language can be used in Pakistan, which can help in not just the screening of neonatal jaundice but also the education of mothers and families. It will be very cost-effective as it requires only a smartphone compared to a transcutaneous bilirubinometer.

To the best of our knowledge, after a thorough literature search, we could not find any available screening program for neonatal jaundice in Pakistan. It had been claimed by another report as well that there is no national screening program for neonatal jaundice early detection. They suggested primary healthcare workers training for the detection of neonatal jaundice. We strongly recommend the initiation of country-wide neonatal jaundice screening programs and educating the public^8^. Providing information to parents and caregivers about the importance of neonatal jaundice screening can increase awareness and encourage them to seek medical attention if they notice any signs of jaundice in their newborn. Healthcare providers, including pediatricians, nurses, and midwives, should receive adequate training on neonatal jaundice screening and management. It will ensure that they can identify and treat the condition early. Establishing and implementing local standardized protocols for neonatal jaundice screening can ensure that all newborns are screened in a timely and consistent manner. Technology-enabled screening solutions, such as mobile apps or telemedicine platforms, can help increase access to screening and follow-up care for newborns, particularly in underserved areas. The illiterate women who are unaware of using smartphones can be helped by their educated partners or neighbors, that can be supportive in contributing to the community’s health. Partnering with community organizations, such as parent groups and advocacy organizations, can help increase awareness about neonatal jaundice screening and improve uptake among families. Advocating for policies that promote universal neonatal jaundice screening and treatment can help improve outcomes for newborns and prevent long-term complications. Regular monitoring and evaluation of neonatal jaundice screening programs can help identify areas for improvement and ensure that the programs are meeting their intended goals. By implementing these strategies, healthcare providers and policymakers can work together to increase awareness and improve outcomes for neonatal jaundice screening. Furthermore, more prospective studies are recommended for neonatal jaundice screening and its’ impact on awareness, education, and the efficacy of smartphone applications.

## Ethical approval

Not applicable.

## Consent

Not applicable.

## Conflicts of interest disclosure

The author(s) received no financial support for the research, authorship, and/or publication of this article.

## Author contribution

H.N., S.O., and K.N.: wrote the manuscript; K.U., M.A.H., and H.B.A.: review-editing; K.U. and S.O.: referencing and formatting.

## Research registration unique identifying number (UIN)

1. Name of the registry: NA.
2. Unique Identifying number or registration ID: NA.
3. Hyperlink to your specific registration (must be publicly accessible and will be checked): NA.

## Guarantor

All authors accept full responsibility for the work and/or the conduct of the study, had access to the data, and controlled the decision to publish.

## Data availability statement

Available upon reasonable request from the corresponding author.

## Provenance and peer review

Not commissioned, externally peer-reviewed.
